# Total glucosides of paeony prevents juxta-articular bone loss in experimental arthritis

**DOI:** 10.1186/1472-6882-13-186

**Published:** 2013-07-21

**Authors:** Chen Chao Wei, Fan Tian You, Li Yu Mei, Sun Jian, Chen Yong Qiang

**Affiliations:** 1 Department of Orthopaedics and Traumatology, Shanghai Municipal Hospital of Traditional Chinese Medicine, Shanghai University of TCM, Shanghai, 200071, China

**Keywords:** Juxta-articular, Osteoporosis, Rheumatoid arthritis, Receptor activator of nuclear factor-B ligand, Total glucosides of paeony

## Abstract

**Background:**

Total glucosides of paeony (TGP) is a biologically active compound extracted from *Paeony* root. TGP has been used in rheumatoid arthritis therapy for many years. However, the mechanism by which TGP prevents bone loss has been less explored.

**Methods:**

TGP was orally administered for 3 months to New Zealand rabbits with antigen-induced arthritis (AIA). Digital x-ray knee images and bone mineral density (BMD) measurements of the subchondral knee bone were performed before sacrifice. Chondrocytes were observed using transmission electron microscopy (TEM). Histological analysis and mRNA expression of receptor activator of nuclear factor-B ligand (RANKL) and osteoprotegerin (OPG) were evaluated in joint tissues.

**Results:**

The BMD value in TGP rabbits was significantly higher compared with that seen in the AIA model rabbits. In addition, the subchondral bone plate was almost completely preserved by TGP treatment, while there was a decrease in bone plate integrity in AIA rabbits. There was less damage to the chondrocytes of the TGP treated group. Immunohistochemical examination of the TGP group showed that a higher percentage of TGP treated chondrocytes expressed OPG as compared to the chondrocytes isolated from AIA treated animals. In contrast, RANKL expression was significantly decreased in the TGP treated group compared to the AIA group. In support of the immunohistochemistry data, the expression of RANKL mRNA was decreased and OPG mRNA expression was enhanced in the TGP group when compared to that of the AIA model group.

**Conclusion:**

These results reveal that TGP suppresses juxta-articular osteoporosis and prevents subchondral bone loss. The decreased RANKL and increased OPG expression seen in TGP treated animals could explain how administration of TGP maintains higher BMD.

## Background

Rheumatoid arthritis (RA) is characterized by chronic inflammation which eventually leads to cartilage degradation and subsequent bone destruction [1]. Bone damage can be either localized, as in juxta-articular bone loss, or more systemic as in generalized bone loss [2]. There are different molecular mechanisms governing systemic bone loss and juxta-articular bone loss [3]. Juxta-articular bone loss mainly occurs in the subchondral bone with synovial tissue invasion of the adjacent cartilage [4]. Although juxta-articular bone loss represents an early feature of RA, very little is known about pathogenesis of juxta-articular bone loss in RA.

Osteoblastic bone formation and osteoclastic bone resorption are involved in the regulation of bone homeostasis [5]. Osteoclasts are the principal instruments of bone destruction. Osteoclasts are regulated through a differentiation process primarily governed by two key cytokines, namely macrophage colony-stimulating factor (M-CSF) and receptor activator of nuclear factor-B ligand (RANKL) [6]. RANKL promotes osteoclast differentiation and plays an important role in the joint destruction seen in arthritis. Osteoprotegerin (OPG) regulates the pro-osteoclastogenic actions of RANKL, which prevents it from binding to and activating RANK.

In arthritis animal models, an imbalance between bone formation and resorption is observed [7]. Recent study have shown that in AIA rabbits, RANKL enhanced osteoclastogenesis may contribute to the development of juxta-articular loss, demonstrating the importance of RANKL in the bone destruction associated with RA [8]. Any imbalance between RANKL and OPG may lead to osteoarticular pathology. In particular, an increase of RANKL and a deficiency of OPG expression results in bone erosion [9,10]. In RA, overexpression of RANKL can induce synovial macrophage differentiation into active osteoclasts, leading to bone destruction. RANKL also plays a key role in the regulation of dendritic cell survival, lymphocyte development, and lymph node organogenesis [11]. The inhibition of bone resorption by the regulation of the RANKL/OPG balance has been demonstrated in postmenopausal women [12]. Treatment with suppressive factor RANKL resulted in an increased amount of bone mineral density (BMD) [13]. High BMD loss in RA patients was associated with joint damage progression, even in the early stages of RA [14]. In summary, inhibition of RANKL expression not only prevents juxta-articular bone loss, but also prevents joint destruction in RA patients.

Non-steroidal anti-inflammatory drugs and biologics are commonly used to alleviate the symptoms of RA [15]. However, there are severe adverse reactions associated with the prolonged use of these drugs [16]. Therefore, it is essential to continue the search for new therapeutic agents to treat RA. In Asia, patients commonly try complementary methods of treatment for RA [17]. Natural plant derived products with therapeutic potential have received substantial attention [18].

Total glucosides of paeony (TGP) is an active compound extracted from the paeony root. TGP is mainly composed of paeoniflorin, with paeoniflorin accounting for approximately 90% of the active constituents of TGP. TGP has been reported to have a clinically significant therapeutic effect on RA, and is becoming more widely used to treat RA [19]. Extensive studies have shown that TGP exhibits anti-inflammatory, analgesic, and immunoregulatory activities [20]. Chang et al. found that TGP exerted its anti-inflammatory effects by inhibiting the production of pro-inflammatory mediators in synoviocytes [21]. Furthermore, Xu et al. showed that the ability of TGP to mediate the levels of IL-1, TNF alpha, IL-6, and PGE in synoviocytes is responsible in part for its inhibition of RA progression [22]. In addition, TGP has been reported to have protective effects on joint destruction. He et al. showed that the protective effect of paeoniflorin might be associated with inhibiting the production of matrix metalloproteinases MMP-1 and MMP-3 by fibroblast-like synoviocytes [23]. These reports have raised the possibility that TGP could prevent juxta-articular osteopenia in RA.

In the present study we investigated the effect of the TGP on juxta-articular osteopenia and subchondral bone destruction. We set out to further our understanding of the mechanisms underlying the therapeutic actions of TGP. In this report we provide experimental evidence to support further development of TGP for clinical use in the treatment of RA.

## Methods

### Drugs and animals

Total glucosides of paeony (TGP) was purchased from Liwah Plant Extraction Technology Co., Ltd Zhejiang Province, China. Indomethacin (IND) was used as a positive control. Each tablet contained 25 mg indomethacin (supplied by Hebei Jizhong Pharmaceutical Co. Ltd., China).

New Zealand rabbits with a body weight of 3–3.5 kg, were obtained from the Shanghai Laboratory Animal Center of the Chinese Academy of Sciences. AIA rabbits were induced as in earlier studies [24]. Briefly, rabbits were injected intradermally with 4 mg ovalbumin (OVA) in Freund’s complete adjuvant (Sigma Chemical Co., St. Louis, MO, USA). This injection procedure was repeated once a week, twice in total. 4 weeks after the last injection of OVA-CFA, the knee joints were injected with 1 ml of OVA once a week. Six weeks after the last booster injection, AIA rabbits were graded by scoring clinical signs according to criteria described previously [25]. The rabbits with a total score of clinical signs higher than six that showed established arthritis were randomly divided into three groups: The AIA model group (*n* = 5), TGP group (*n* = 5) and IND group (*n* = 5). The normal group rabbits (*n* = 5) did not undergo a procedure. All rabbits were housed under standard laboratory conditions. The rabbits in the TGP group received intragastric (i.g.) administrations of TGP at 60 mg/kg per day. Rabbits in the IND group received i.g. administrations of IND at 3 mg/kg per day. Both dosages are equivalent to 10 times the clinical dosage for a 60 kg adult. The duration of drug treatment was for a period of 3 months. Rabbits in the normal and AIA model groups received the same volume of normal saline. Rabbits were anaesthetized and sacrificed after 3 months. Articular cartilage and subchondral bone were collected from knee joints of rabbits after being euthanized. This protocol received approval by the committee for Animal Experiments of the University Medical Centre, Shanghai, China.

### Radiographic analysis and bone mineral density measurements

Before being euthanized, radiographic analysis and bone mineral density measurements were used to determine the presence of juxta-articular osteoporosis in rabbit knees. All radiographs were using the conventional microradiography system (Softex, Tokyo, Japan), at 40 kV and 3 mA for 75 s. Rabbits were placed in the supine position and the x-ray beam was centered over the femorotibial joints [26]. To measure the BMD of rabbit’s juxta-articular bone, all rabbits underwent dual energy x-ray absorptiometry scanner (DEXA, Hologic Inc., Waltham, MA). Rabbits were placed in the supine position and the knee joint was measured by BMD. BMD measurements were conducted on the medial femoral condyles and tibial plateaus.

### Histological assessment of cartilage destruction and subchondral bone thickness

The proximal tibia was cut into 5 μm thick frontal sections for histomorphometry. The sections were stained with Safranin O-fast green. The Mankin grading system was used to evaluate cartilage destruction by two independent pathologists [27]. The width of the subchondral bone plate was measured between the tidemark of articular cartilage and subchondral bone ends. Subchondral bone plate thickness was evaluated in three different regions of the femur condyle at × 100 magnification.

### Electron microscopy

After demineralization, tissue specimens were placed in 2.5% glutaraldehyde in Sorenson’s phosphate buffer and rinsed in buffer and decalcified in a controlled manner using 10% EDTA. We then used an increasingly concentrated series of ethanol solutions to dehydrate. Each specimen was embedded in Epon812 for transmission electron microscopy (TEM). Tissue sections were cut to 60 nm thickness and subsequently double stained sequentially using uranyl and then phosphotungstic acid. We then performed TEM to observe chondrocytes (HITACHI H-500, Japan).

### Immunohistochemical analysis of RANKL and OPG expression in subchondral bone

5 μm thick sections were used for immunohistochemical examinations. The sections were incubated with primary antibodies for OPG (1:50 dilution) and RANKL (1:100 dilution0 [Santa Cruz]. After staining the nucleus with DAPI, the slices were then stained with Cy3-labeled secondary antibodies for 1 h at 37°C. Negative control staining was performed using PBS instead of the primary antibody.

### Quantitative real time PCR analysis of RANKL mRNA and OPG mRNA

Total RNA was extracted from subchondral bone and cartilage in the tibiae articular using Trizol reagent. Two microliters of each sample was used for real-time PCR in a Rotor-Gene 2000 system (Australia), which was performed as described previously [28]. Relative quantitation was calculated using delta cycle threshold relative quantitation. Specific primer sequences for PCR were designed as follows:

RANKL: Forward primer 5′-TTTGCAGGACTCGACTCTGGAG

Reverse primer 5′-TCCCTCCTTTCATCAGGTTATGAG;

OPG: Forward primer 5′-ATCATTGAATGGACAACCCAGG

Reverse primer 5′-TGCGTGGCTTCTCTGTTTCC;

GAPDH: Forward primer 5′-GATCGTGGAAGGGCTAATGA

Reverse primer 5′-GACTTTGCCTACAGCCTTGG.

### Statistical analysis

All results were expressed as mean and standard deviation (SD). Measurement data were analyzed using a one-way analysis of variances (ANOVA, SPSS 11.0). Ridit analysis was used in order to rank data. A result of *p* < 0.05 was considered to be statistically significant.

## Results

### Effect of TGP inhibition in juxta-articular osteopenia

**Figure 1:**
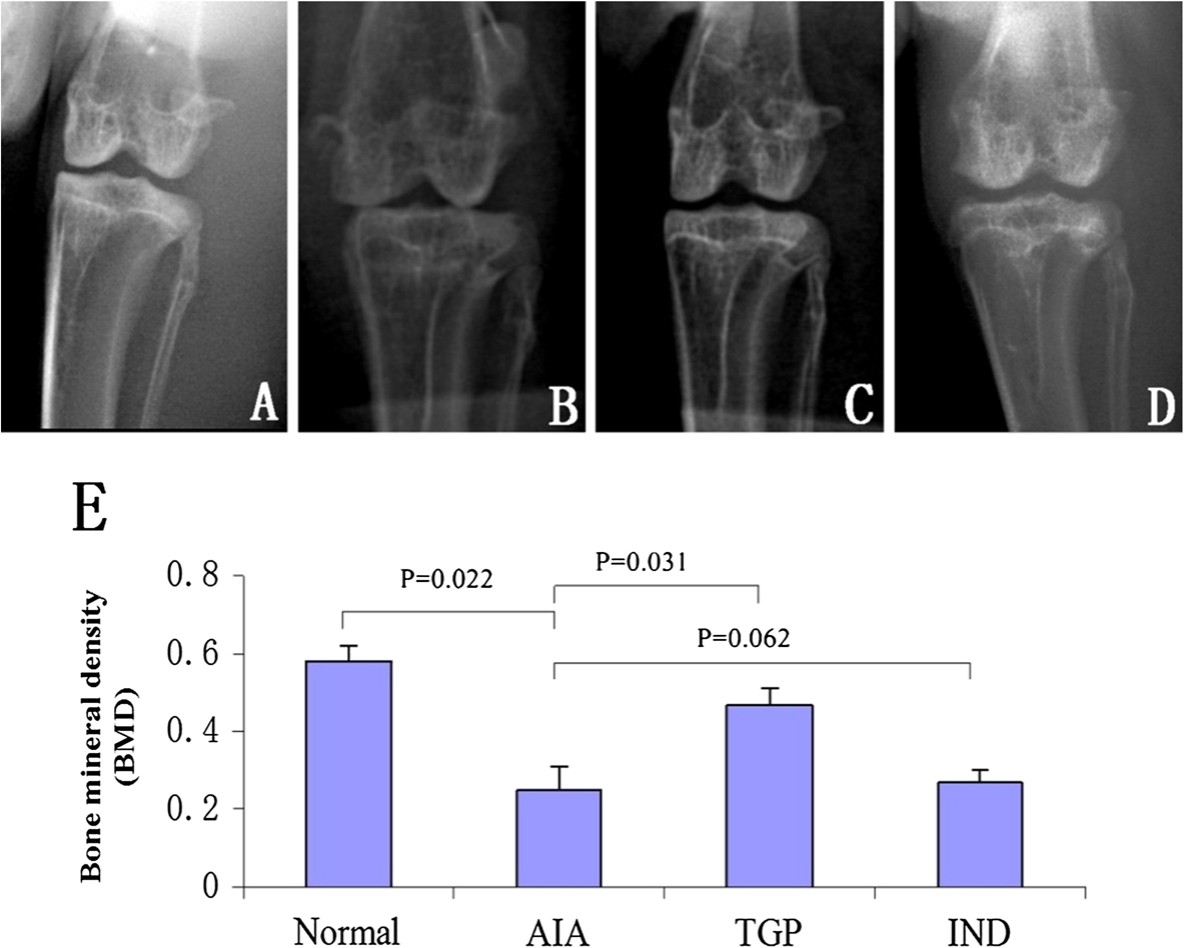
**Representative X**-**ray photographs of knee bone and BMD of knees in each group of rabbits. (A)** Normal group **(B)** AIA group **(C)** TGP group **(D)** IND group **(E)**: Densitometric analysis of bone mineral density (BMD, g/cm^2^) in the subchondral bones for each group of rabbits. Results are presented as mean ± S.D.

The AIA group rabbits exhibited juxta-articular bone osteopenia and the joint space was narrowed in the knees as evidence by radiographic analysis. However, less bone loss occurred in the TGP treated group (Figure 1). The BMD was lower in AIA rabbits as compared to healthy controls. The difference in BMD between AIA and control rabbits reached statistical significance (*p* = 0.022). However, the BMD value in TGP treated rabbits was significantly higher compared with that in the untreated AIA group (*p* = 0.031). Furthermore, we also observed that the IND positive control group had a higher BMD than AIA rabbits, although this result did not attain statistical significance (Figure 1E).

### TGP reduces arthritis cartilage and subchondral bone damage

**Figure 2:**
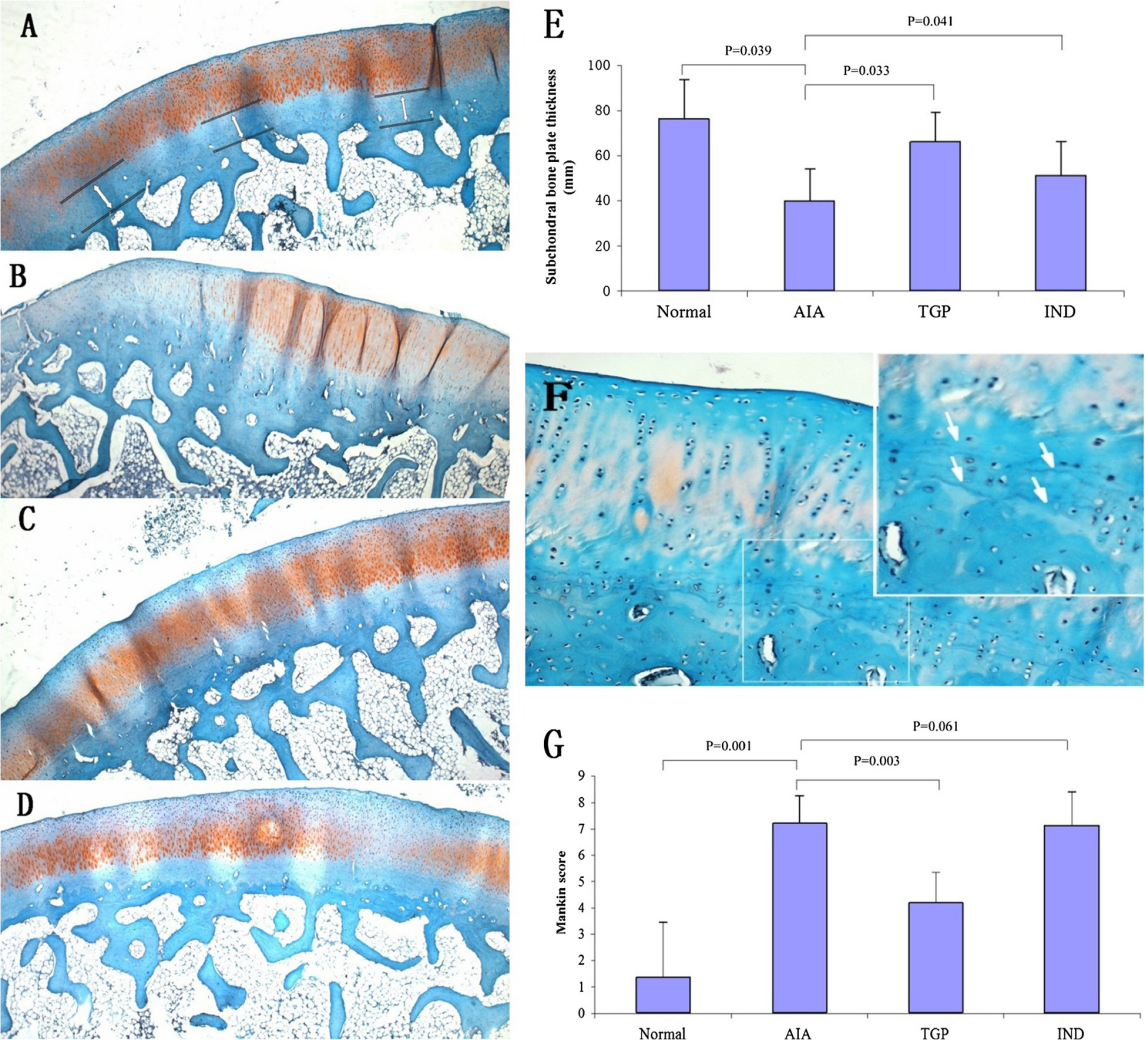
**Histological evaluation of juxta**-**articular subchondral bone plate thickness and cartilage damage.** Safranin O-fast green stained sections of subchondral bone **(A)** Normal group. The width of the subchondral bone measurements was measured in three regions of the femur condyle. **(B)** AIA group **(C)** TGP group **(D)** IND group (original magnification × 100). **(E)** Subchondral bone plate thickness in each group of rabbits. Results are presented as mean ± S.D. **(F)** The tidemark is duplicated in TGP cartilage (original magnification × 300). **(G)** Mankin score of the rabbit cartilage in each group. Results are presented as mean ± S.D.

Results showed that in the AIA group, there was a loss of Safranin O-fast green staining accompanying cleft formation. In addition discontinuous bone trabeculae were present in the subchondral bone and blood vessel formation occurred within the osteochondral junction. We have observed a decrease in the thickness of the subchondral bone plate compared with those healthy ones (Figure 2). This difference in subchondral bone plate thickness reached statistical significance (*p* = 0.039). However, the subchondral bone plate was almost completely preserved in the TGP treated groups, and was partially preserved in the IND group. Quantitative evaluation of the width of the subchondral bone plate showed a significant difference between TGP treated groups and the AIA groups (*p* = 0.033) (Figure 2E). Furthermore, the tidemark was duplicated in TGP treated cartilage (Figure 2F). The Mankin score was significantly higher in AIA rabbits (*p* = 0.001) as compared to that exhibited in healthy animals. There was a significantly lower Mankin score in the TGP treated group compared with the AIA group (*p* = 0.003), and no statistically significant differences in the Mankin score between the IND and AIA group (*p* = 0.061) (Figure 2G).

### Effect of TGP on chondrocytes

**Figure 3:**
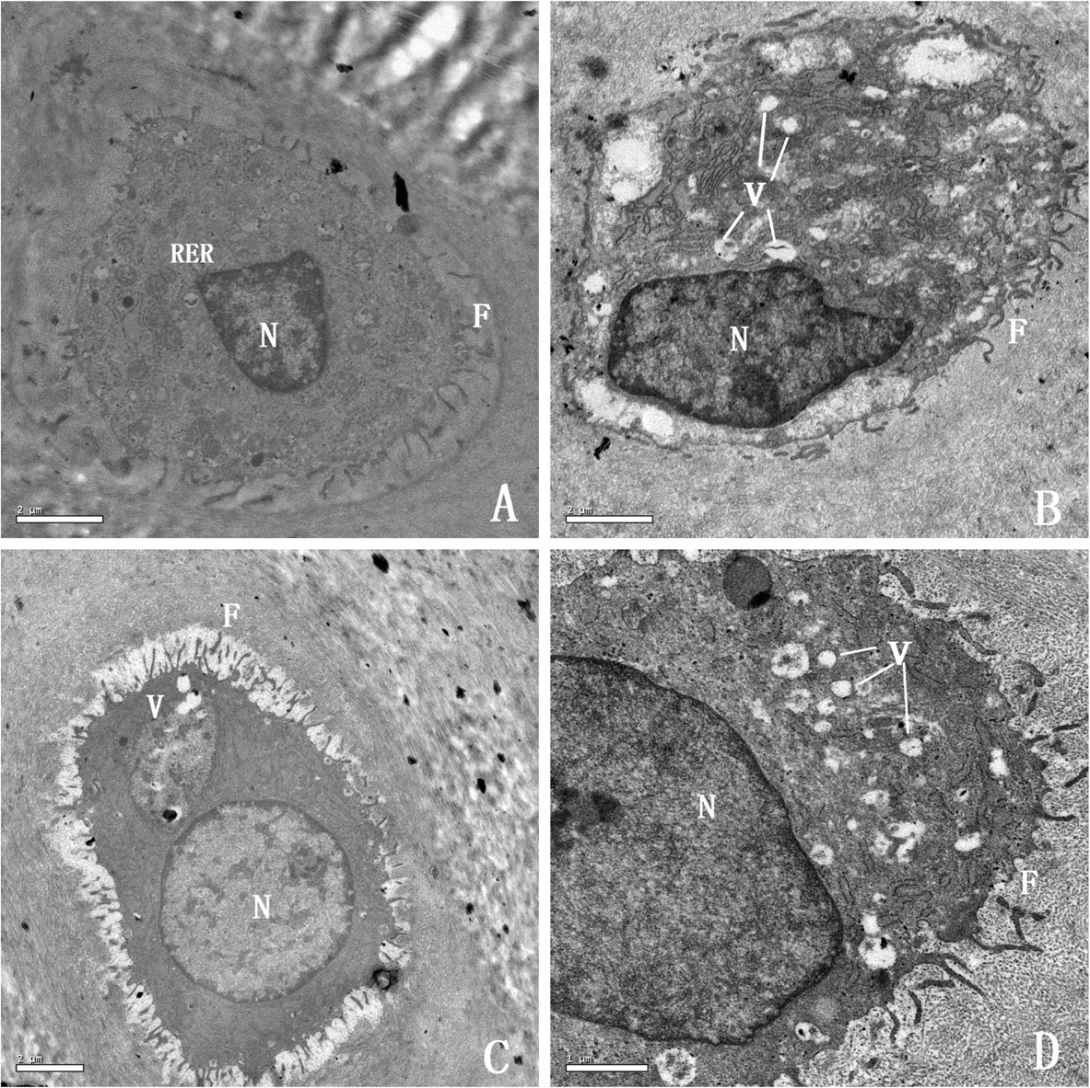
**TEM micrographs of chondrocytes. (A)** Normal group. Healthy normal chondrocytes contain a euchromatic nucleus, very abundant rough endoplasmic reticulum, and long filopodia (× 10,000 magnification). **(B)** AIA group. AIA chondrocytes exhibit short filopodia and show a reduction in the number of Golgi bodies and rough endoplasmic reticulum. **(C)** TGP group. TGP chondrocytes have a strong reduction of the number of vacuoles. The fibrosis on the surface of the cartilage was larger and wider (× 15,000). **(D)** IND group. Chondrocytes from this group had an abundance of vacuoles, similar to what was seen in the AIA group (× 20,000) (F, filopodia; N, euchromatin nucleus; RER, rough endoplasmic reticulum).

Representative TEM micrographs of chondrocytes are shown in Figure 3. At the end of the 3th mouth, we observed a normal cytoplasm contains a euchromatic nucleus. A region of their plasma membranes bears some relatively long filopodia and there were abundant rough endoplasmic reticulum, numerous well shaped Golgi bodies and mitochondria located in the cytoplasm. Chondrocytes in the AIA group displayed a different ultrastructure. AIA chondrocytes displayed a region of plasma membrane which bears some relatively short filopodia, typical vacuoles were abundant in the cytoplasm whereas rough endoplasmic reticulum, Golgi bodies and mitochondria were strongly reduced. However, in the TGP treated group, TEM micrographs of deep zone chondrocytes showed that their cytoplasms contain a euchromatic nucleus. A strong reduction of the number of vacuoles and an abundant presence of rough endoplasmic reticulum, well shaped Golgi bodies, and mitochondria were observed in TGP chondrocytes. The filopodia present on the surface of the chondrocytes were larger and wider in the TGP group compared with that in the AIA group.

### Inhibition of RANKL and enhanced OPG expression in cartilage are associated with TGP therapy

**Figure 4:**
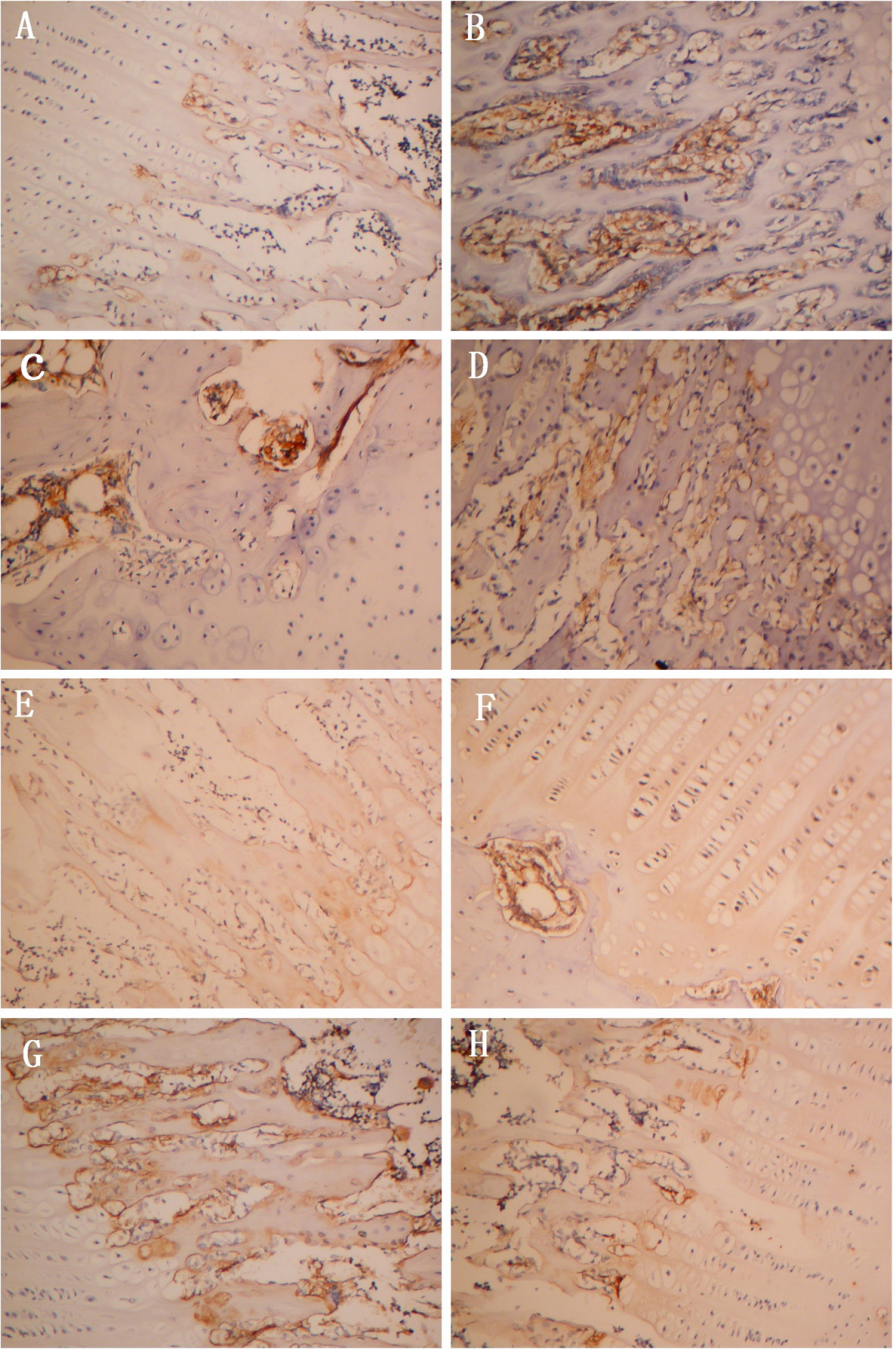
**RANKL and OPG expression in articular cartilage and subchondral bone.** Histological sections were submitted for immunohistochemical analyses. RANKL staining is shown in each group in Figure 4**(A-D)**: **(A)** Normal group. RANKL immunolabeling was rarely detected. **(B)** AIA group. RANKL immunolabeling was detected at high levels, especially in subchondral bone. **(C)** TGP group. TGP decreased RANKL expression. **(D)** IND group. OPG staining for each group is shown in Figure 4**(E-H)**: **(E)** Normal group. OPG was expressed only in deep zone. **(F)** AIA group **(G)** TGP group. OPG was highly expressed in subchondral bone. **(H)** IND group (original magnification × 400).

Immunohistochemical detection showed that RANKL and OPG expression were expressed in very few cells in normal group cartilage. However, AIA rabbits exhibited higher expression of RANKL with higher intensity throughout the articular cartilage, with an increase in intracellular RANKL expression in, both cartilage and subchondral bone. There were very few cells expressing OPG in AIA rabbit cartilage. In the TGP group, RANKL expression was significantly decreased in the cartilage. Nevertheless, OPG expression had higher positive staining compared with that seen in the AIA group. In the cartilage of IND rabbits, an increase in the intensity of the RANKL staining was observed (Figure 4).

**Figure 5:**
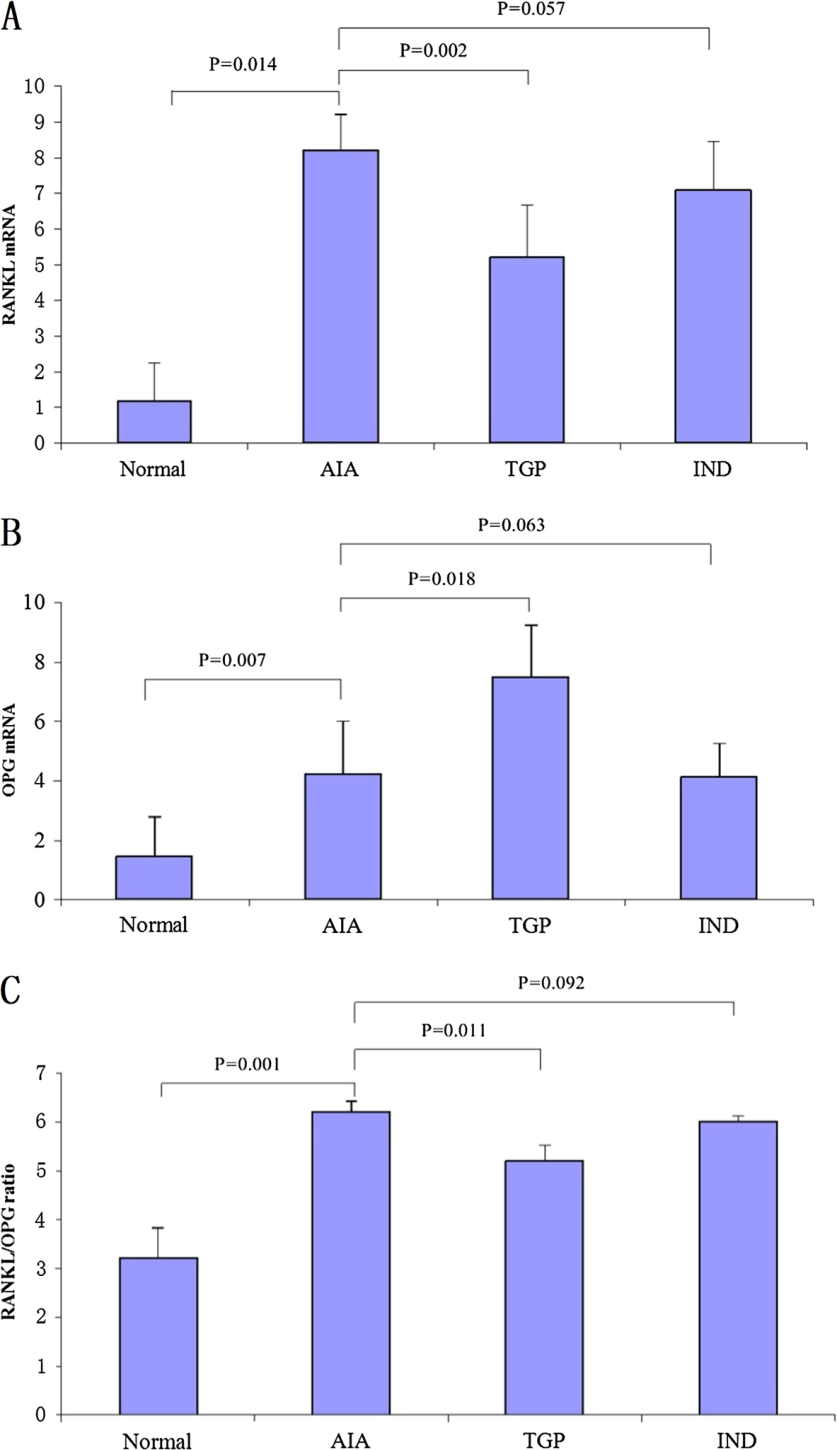
**TGP treatment decreases RANKL expression and increases OPG expression. (A)** RANKL mRNA and **(B)** OPG mRNA expression in each experimental group. **(C)** RANKL/OPG ratio. Results are presented as mean ± S.D.

Quantitative RT PCR analysis indicated that the expression of RANKL and OPG mRNA species are low in the normal group, while the expression of RANKL increases significantly in the AIA group in comparison with that of normal group (*p* = 0.014); The expression of RANKL mRNA in the TGP treated group was significantly decreased. In contrast, an enhanced expression of OPG mRNA was expressed in the TGP group as compared with that in AIA group after administration (*p* < 0.01). Although the RANKL mRNA level in IND group tended to be reduced by IND treatment as compared with that in AIA group, there was no significant difference (*p* = 0.057) (Figure 5A, B). Moreover, when compared with normal group, the RANKL/OPG ratio in articular cartilage showed an increase in AIA group (*p* = 0.001). An increased ratio, however, was not found in TGP group when compared with AIA group (*p* = 0.011). In addition, the RANKL/OPG ratio between the IND group and AIA group, there was no significant difference (*p* = 0.092) (Figure 5C).

## Discussion

Rheumatoid arthritis is a systemic autoimmune inflammatory disease involving the breakdown of cartilage and juxta-articular bone. Patients with RA have irreversible bone destruction and a poor functional outcome [29]. Therefore, preventing bone destruction is important for RA therapy. In this study, we demonstrated that TGP has an inhibitory effect on juxta-articular osteopenia and subchondral bone destruction.

The subchondral bone plays a crucial role in the pathogenesis of RA [30]. In our study, TGP fed rabbits maintained higher BMD in the juxta-articular space compared with AIA rabbits. These findings are of great interest considering juxta-articular bone destruction represents an early feature of RA [31]. However, the pathogenesis of subchondral bone destruction in RA has not yet been fully explored. The subchondral bone consists of the subchondral bone plate and the subarticular spongiosa. The tidemark is the boundary between the deep cartilage layer and the calcified cartilage layer, and is visible as a basophilic line [32]. In RA, the disruption of the tidemark produces cross-talk between the subchondral bone and cartilage [33]. Articular chondrocytes near the joint surface will produce factors such as vascular endothelial growth factor, which may aggravate subchondral tissues and other locations in the joint [34]. In this study, we also found the development of blood vessels within the osteochondral junction in AIA rabbits. The presence of blood vessels beyond the tidemark may contribute to bone erosion.

The protective barrier between intra-articular and subchondral compartments was removed by loss of osteochondral integrity. Other investigators have shown that osteochondral angiogenesis and channels invading the cartilage in RA have been associated with nerve growth factor expression. Nerve growth factor induces functional and structural changes within the cartilage that may contribute to the pain associated with RA [35]. Therefore, treatments targeted at the subchondral bone have the potential to improve palliative care of RA patients. In this study, we found that the tidemark is duplicated in TGP group cartilage. This abnormality indicates that the mineralization process took place in the cartilage chondrocytes. Chondrocyte hypertrophy and extracellular matrix mineralization may result in tidemark duplication.

In the present study, the TEM micrographs of arthritis cartilage showed degeneration in the chrondrocytes from the AIA group and failure to develop significant numbers of important cytoplasmic organelles. The degenerate chondrocytes showed a strong reduction in cytoplasmic rough endoplasmic reticulum, Golgi bodies, and mitochondria. Previous studies showed that the degeneration of chondrocytes was caused by a deficiency in Golgi apparatus development, which could be in charge of sugar synthesis and protein transport in chondrocytes [36]. However, in the TGP rabbits, there was less damage in the chondrocytes. This result suggests that inhibition of chondrocytes degeneration might be one of the important mechanisms by which TGP prevents juxta-articular bone destruction.

Interleukin-17 (IL-17) plays a vital role in RANKL induction, osteoclastogenesis and bone erosion. IL-17 is produced mainly by pro-inflammatory T-helper subset (Th17) cells and both IL-17 and Th17 cells are deeply implicated with the pathogenesis of RA [37]. IL-17 enhances local inflammation and increases the production of inflammatory cytokines, such as tumor necrosis factor (TNF)-α, IL-1, and IL-6, which further promote RANKL expression and activity [38]. In previous studies, it has been confirmed that TGP treatment significantly decreased percentage and number of Th1 and Th17 cells in collagen induced arthritis [39]. Moreover, treatment with TGP decreased expression of T-bet as well as phosphorylation of signal transducer and activator of transcription 1 (STAT1) and STAT3 [39].

To further understand the mechanisms underlying protective effects of TGP against the development of arthritis, we evaluated the expression of RANKL and OPG. Previous studies have associated osteoblast maturation with gene expression levels of RANKL and OPG [40]. RANK and RANK ligand factors are critical for controlling osteoclast differentiation and bone resorption. RANK and RANKL may be implicated in subchondral bone alterations in RA [41]. Thus, abnormally high levels of RANKL are regarded as good biomarkers for pathogenesis of RA bone damage [42]. In this study, the expression of RANKL was higher in the articular cartilage of rabbits with AIA. After administration of TGP, we found lower expression of RANKL, and higher expression of OPG compared to those seen in AIA rabbits. These results suggested that reduced levels of RANKL, along with increased OPG levels could lead to a decrease in osteoclastogenesis and osteoclast activity, which results in inhibition of osteoclastic bone resorption and eventually higher bone mass and BMD in TGP fed rabbits. The results show that action of TGP on regulating juxta-articular osteoporosis might be through inhibiting expression of RANKL, thus altering the RANKL/OPG ratio.

## Conclusions

The overall results revealed for the first time that TGP could suppress juxta-articular osteoporosis and prevent subchondral bone destruction. This study also determined that TGP maintains a higher BMD in part due to altering the RANKL/OPG ratio.

## Abbreviations

TGP: Total glucosides of paeony; AIA: Antigen-induced arthritis; BMD: Bone mineral density; RANKL: Receptor activator of nuclear factor-B ligand; OPG: Osteoprotegerin; TEM: Transmission electron microscopic; IND: Indomethacin; IL-17: Interleukin-17; Th17: T-helper subset; STAT1: Signal transducer and activator of transcription 1.

## Competing interests

The authors declare that they have no competing interests.

## Authors’ contributions

CCW carried out the study; FTY and CYQ designed the experiments. LYM wrote the manuscript; all authors read and approved the final manuscript.

## Pre-publication history

The pre-publication history for this paper can be accessed here:

http://www.biomedcentral.com/1472-6882/13/186/prepub
